# Saffron extract and crocin exert anti-inflammatory and anti-oxidative effects in a repetitive mild traumatic brain injury mouse model

**DOI:** 10.1038/s41598-022-09109-9

**Published:** 2022-03-23

**Authors:** Marwa Salem, Mariam Shaheen, Abeer Tabbara, Jamilah Borjac

**Affiliations:** 1 Department of Biological Sciences, Beirut Arab University, Debbieh, Lebanon; 2 Department of Pathology and Laboratory Medicine, American University of Beirut, Beirut, Lebanon; 3 Department of Biological Sciences, Beirut Arab University, Riad El Solh, P.O. Box 115020, Beirut, 11072809 Lebanon

**Keywords:** Biochemistry, Molecular biology, Physiology, Neurology

## Abstract

Saffron *Crocus sativus* L. (*C. sativus*) is a flower from the iridaceous family. Crocin, saffron’s major constituent, and saffron have anti-oxidative and anti-inflammatory activities. In this work, the neuroprotective effects of saffron and crocin are being investigated in a repetitive mild traumatic brain injury (rmTBI) mouse model. A weight drop model setup was employed to induce mild brain injury in male albino BABL/c mice weighing 30–40 g. Saffron (50 mg/kg) and crocin (30 mg/kg) were administrated intraperitoneally 30 min before mTBI induction. Behavioral tests were conducted to assess behavioral deficits including the modified neurological severity score (NSS), Morris water maze (MWM), pole climb test, rotarod test, and adhesive test. The levels of TNF alpha (TNF-α), interferon-gamma (IFN-γ), myeloperoxidase activity (MPO), malonaldehyde (MDA), and reduced glutathione (GSH) were measured. Histological analysis of different brain parts was performed. Both saffron and crocin demonstrated marked improved neurological, cognitive, motor, and sensorimotor functions. Besides, both compounds significantly reduced the oxidative stress and inflammatory processes. No abnormal histological features were observed in any of the injured groups. Saffron extract and crocin provide a neuroprotective effect in a mouse model of rmTBI by decreasing oxidative stress, inflammatory responses, and behavioral deficits.

## Introduction

Mild brain injuries (mTBI) or concussions encountered in car accidents or falls have become of great concern especially due to their associated consequences at the behavioral, neurological, and cognitive levels. Negative results on routine neuroimaging such as computed tomography (CT) have made it difficult to clearly define a concussion and the most recent definition stated it as “a complex pathophysiological process affecting the brain, induced by traumatic biomechanical forces”^1^. According to the American Congress of Rehabilitation Medicine (ACRM), mTBI patients should exhibit at least one of the following symptoms: (1) loss of consciousness for no more than 30 min, (2) loss of memory, (3) immediate mental changes such as dizziness, disorientation or confusion, (4) short or long-term neurological deficits^2^. Each year, around 50 million people have a traumatic brain injury (TBI) each year with 70–90% sustain mTBI^3^. Individuals are more likely to be susceptible to a new injury if they had sustained two before as shown by a cohort study performed by the National Athletic Association NCAA, on 4251 players, suggesting that the likelihood to have a future concussion increases with players having a history of insults that slow down their recovery time^4^. Universally, the number of TBI patients is on the rise^5^. Each year, around 69 million people suffer from TBI and its related outcomes (death and disability)^6^. Effects depend on injury severity and time interval between hits; so, a temporal window exists during which the concussed brain is more prone to a subsequent concussion as revealed by experiments done on rodents^7^. Interestingly, the longer the inter-hit interval time, the more protection against long-term deficits is conferred^8^. Given the fact that mTBI is a recoverable process, in which patients may not show any impairment sign in the short-term, it does not exclude the long-term detrimental neurological sequelae. There is growing evidence of its association with developing neurological disorders later in life such as Alzheimer’s disease (AD), Parkinson’s disease (PD), chronic traumatic encephalopathy (CTE)^9^. Rodent models have explored the cumulative effects of rmTBI on the cognitive performance that is similar to clinical studies^10^. Angoa-Perez et al., showed that the neurological consequences of repeated mild brain injuries were more severe than single brain injuries where cognitive deficits were manifested in the Morris water maze (MWM) without presenting brain cell loss^11^. After TBI, a series of events are initiated to boost the inflammatory reaction such as increased neural cell death through glial cell activation, and recruitment of peripheral immune cells such as neutrophils and monocytes that can cross the blood–brain barrier^12,13^. Once glial cells are activated, they start secreting cytokines such as tumor necrosis factor-α (TNF-α), inflammasomes (NLRP), nuclear factor kappa-light-chain-enhancer of activated B cells (NF-κB) interleukin-1β (IL-1β), and interferon-γ (IFN-γ) that control injury progress and tissue recovery post-TBI^14^. After brain injuries, cytokines are discharged to get tangled in different body responses such as the autonomic, metabolic, and behavioral responses (cognitive and motor responses)^15^. Furthermore, many potentially noxious compounds that generate oxidative stress through the production of reactive oxygen species (ROS) are released^16^. These pro-inflammatory and cytotoxic factors are harmful to neurons and provoke further activation of microglia, leading to progressive degeneration of dopaminergic neurons that regulate motor and cognitive function^17^. Recently there has been a great interest in investigating, the neuroprotective role of natural compounds as neuroprotective drugs in TBI. Saffron and its metabolite crocin have been shown to possess diverse biological activities such as anti-inflammatory, antioxidative, and anti-apoptotic^18^. Studies have proved crocin improved cognitive performance in the ischemia/reperfusion (I/R) injury model^19^, against intracerebroventricular streptozotocin-induced spatial memory deficit^20^. Saffron was also found to improve cognitive disorders in Alzheimer's disease in an animal model^20,21^. To the best of our knowledge, only one study has investigated the effect of crocin on TBI in a mouse model of controlled cortical impact (CCI) demonstrating its neuroprotective role through improved brain edema, decreased microglial activation, and decreased cell apoptosis 24 h post-injury^22^. We aim, in this study, to investigate the neuroprotective role of saffron extract and crocin in a repetitive mild traumatic brain injury (rmTBI) mouse model through behavioral analysis, histological study and measurement of oxidation and inflammation markers. Promising results might help design therapeutic interventions especially for athletes and people who frequently encounter brain concussions.

## Results

### Bodyweight change

**Figure 1:**
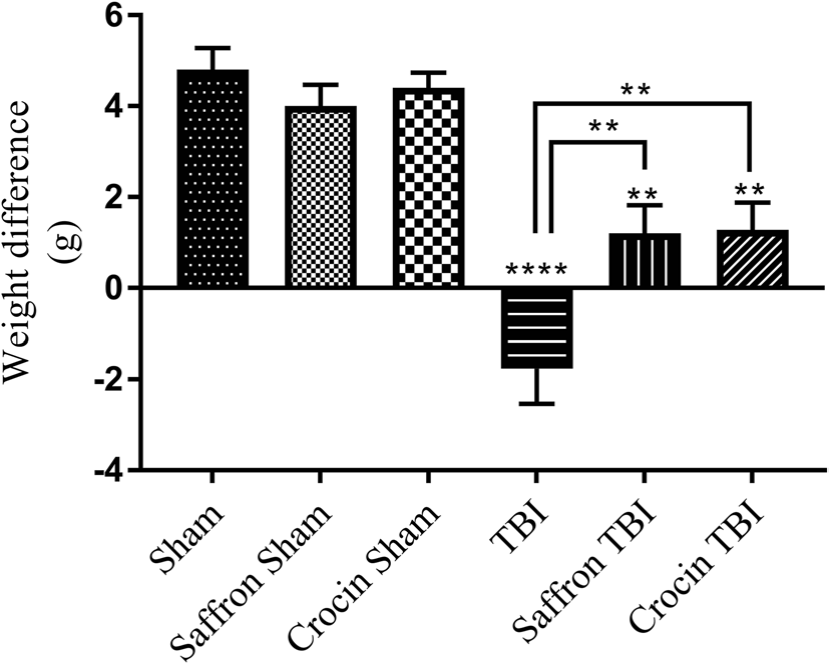
The weight difference between day 0 and day 14. Data are mean ± SEM of 25 mice per group. TBI group showed a negative difference (p < 0.0001). Both saffron TBI group and crocin TBI showed a positive difference (p < 0.0021).

Weights of all mice (n = 25 per group) were followed over the 14 days and the difference between day 1 and day 14 was analyzed (Fig. 1). In control groups, i.e., sham, saffron sham, and crocin sham, the weights increased with an average of 4 g. However, in the untreated TBI group, mice significantly lost 1.7 g (p < 0.0001). Upon treatment with saffron extract and with crocin, mice significantly regained weight (1.2 g on average, p < 0.0021) implying that both are capable to prevent weight loss associated with the induced trauma. These results showed that the injury affects mice weight and both saffron and crocin treatments were powerful in ameliorating weight loss related to this injury.

### Neurological severity score

**Figure 2:**
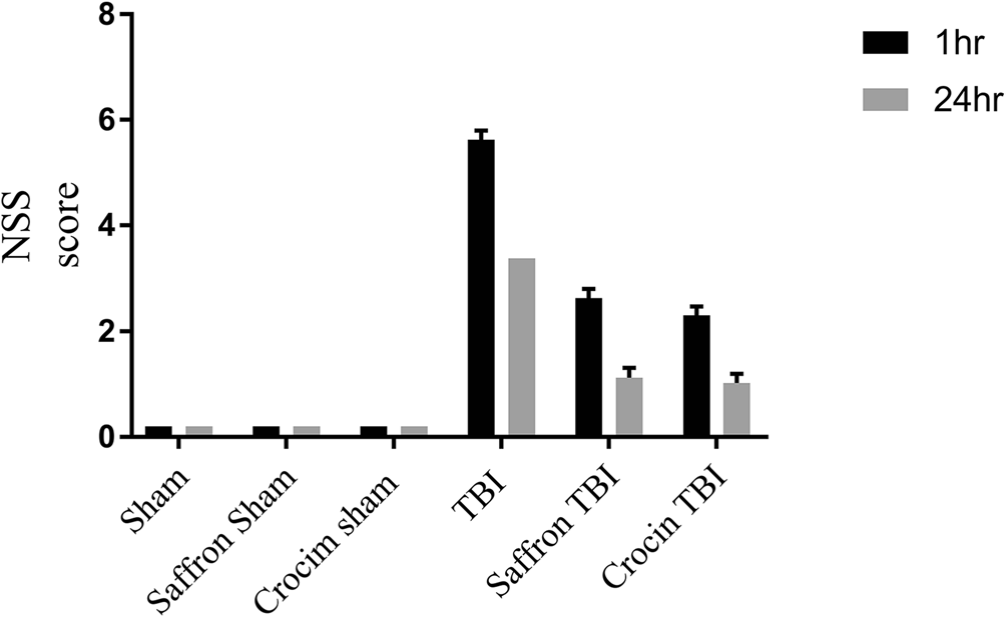
Neurological severity score (NSS) for mice in all experimental groups. Data obtained showed that the TBI group presented higher NSS than the sham group. Saffron and crocin treatment reduced the NSS score. Data are mean ± SEM of eight mice per group.

The NSS was assessed at 1 h and 24 h post the final injury (Fig. 2). The NSS of all sham animals is 0 NSS points. Mice were able to perform completely all tasks at the two-time points proving their normal neurological behavior. However, after 1 h, the TBI group recorded an NSS of 5 indicating the occurrence of mild trauma, and it dropped to 1 after 24 h. As for the saffron TBI and crocin TBI groups, the initial NSS recorded was 3 and dropped to zero after 24 h indicating the restoration of normal neurological behavior after saffron extract and crocin treatment.

### Behavioral assessment

#### Effect of crocin and saffron extract on motor function after TBI

**Figure 3:**
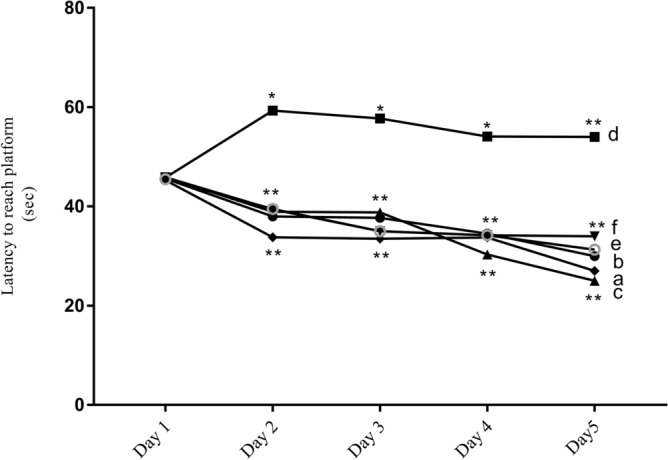
Morris water maze results of all experimental groups following TBI. Short-term defects in spatial learning were detected in the TBI group. Saffron and crocin enhanced the induced cognitive deficits and both groups showed no difference in their latency to reach the platform a: sham, b: saffron sham, c: crocin sham, d: TBI, e: saffron TBI, f: crocin TBI. Data are mean of eight mice per group. (*) and (**) represent p < 0.03 and < 0.0021 respectively.

MWM was used to assess the spatial learning deficits after TBI. The latency to find the platform was measured in all groups (n = 8). Only TBI mice exhibited cognitive dysfunction and needed 20 s more than the sham to locate the platform (Fig. 3, line d). Treating TBI mice with saffron extract and with crocin allowed them to spot the platform faster by 20 s compared to untreated TBI mice as shown in Fig. 3 (line e and f) (p < 0.0021). It is important to note these treatments induced similar results compared to sham groups.

#### Effect of crocin and saffron extract on motor and sensorimotor function

**Figure 4:**
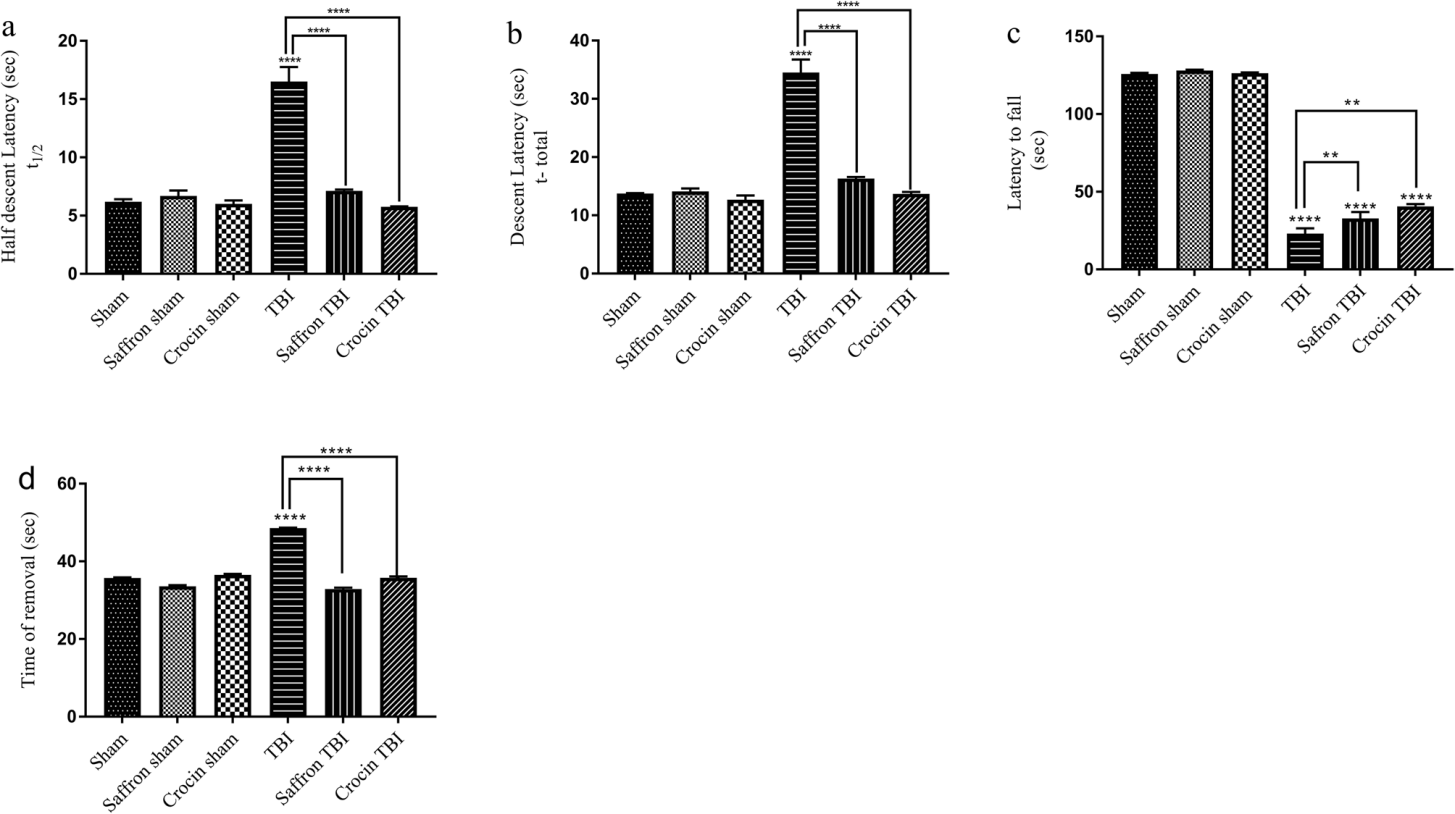
Pole climb, rotarod, and adhesive tests results. (**a**, **b**) show t1/2 and t values respectively in pole climb test results. Data is mean ± SEM of eight mice per group. (****) represents p < 0.0001. (**c**) The latency to fall from the rotarod in (s). The latency was analyzed for all experimental groups. Data is mean ± SD of eight mice per group. (**) and (****) represent p < 0.0021 and < 0.0001 respectively. (**d**) The latency to remove the tape in adhesive test for all experimental groups. Data are mean ± SEM of eight animals in each group. (****) represents p < 0.0001.

To assess motor and sensorimotor functions, pole climb, rotarod, and adhesive tests were performed as shown in Fig. 4. In the pole climb test, the latency of the mouse to cross half the distance (t_1/2_) of the rod and the time to reach the bottom (t) were recorded (Fig. 4a,b correspondingly). Control mice exhibited similar behavioral results, with an average t_½_ of ∼ 6 s and t of ∼ 14 s. After the injury, a significant increase in t_1/2_ and t was observed in the TBI group of 16 and 34 s respectively. TBI mice treated with saffron extract or crocin showed better performance. The t_1/2_ and t values decreased to 7 and 16 s with saffron treatment, and to 7 and 14 s with crocin treatment respectively (p < 0.0001), reaching values close to shams and thus reflecting the protective effects they exert on motor outcomes.

In the rotarod test, the control groups; sham, saffron sham, and crocin sham, recorded approximately 126 s on the rotarod during the day of the performance (Fig. 4c). However, the latency of the TBI group on the rotarod decreased significantly to 23 s (5.5 folds, p < 0.0001). Although the injury worsened the motor coordination skills, saffron and crocin treatments were able to improve the motor function by increasing the latency by 9 and 17 s respectively (1.7 and 1.4 folds with respect to TBI respectively p < 0.0021).

The sensorimotor deficits were assessed by the adhesive tape removal test (Fig. 4d). The time needed to remove the adhesive tape was comparable among the shams (~ 36 s). Additional 13 s were needed by TBI mice to remove the tape (p < 0.0001). Both saffron extract and crocin treatment were able to minimize significantly this time close to the normal group records (p < 0.0001).

Hence, the primary endpoint of the behavioral analysis showed that rmTBI induced cognitive and sensorimotor deficits that were reduced remarkably after treatment.

### Histology

**Figure 5:**
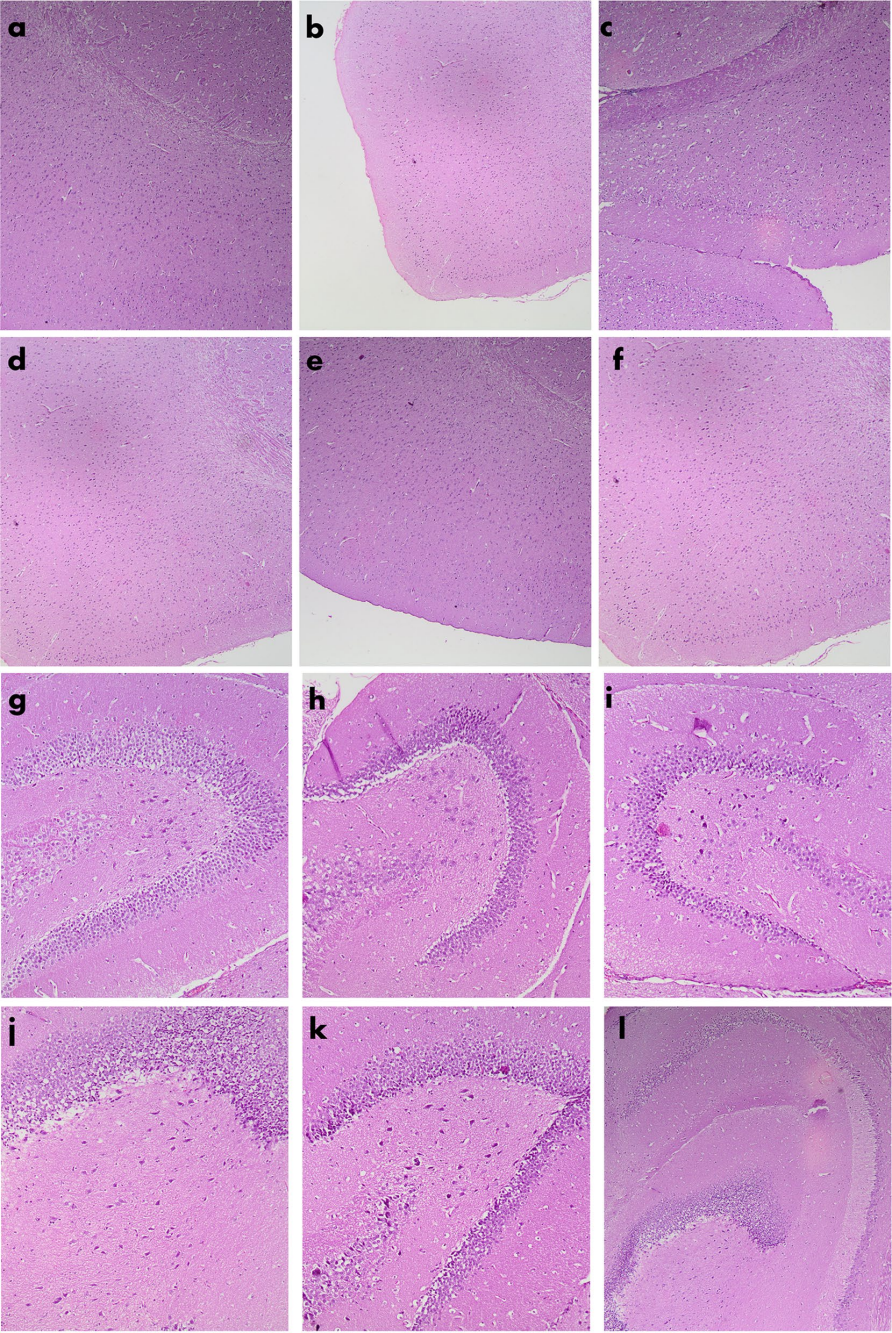

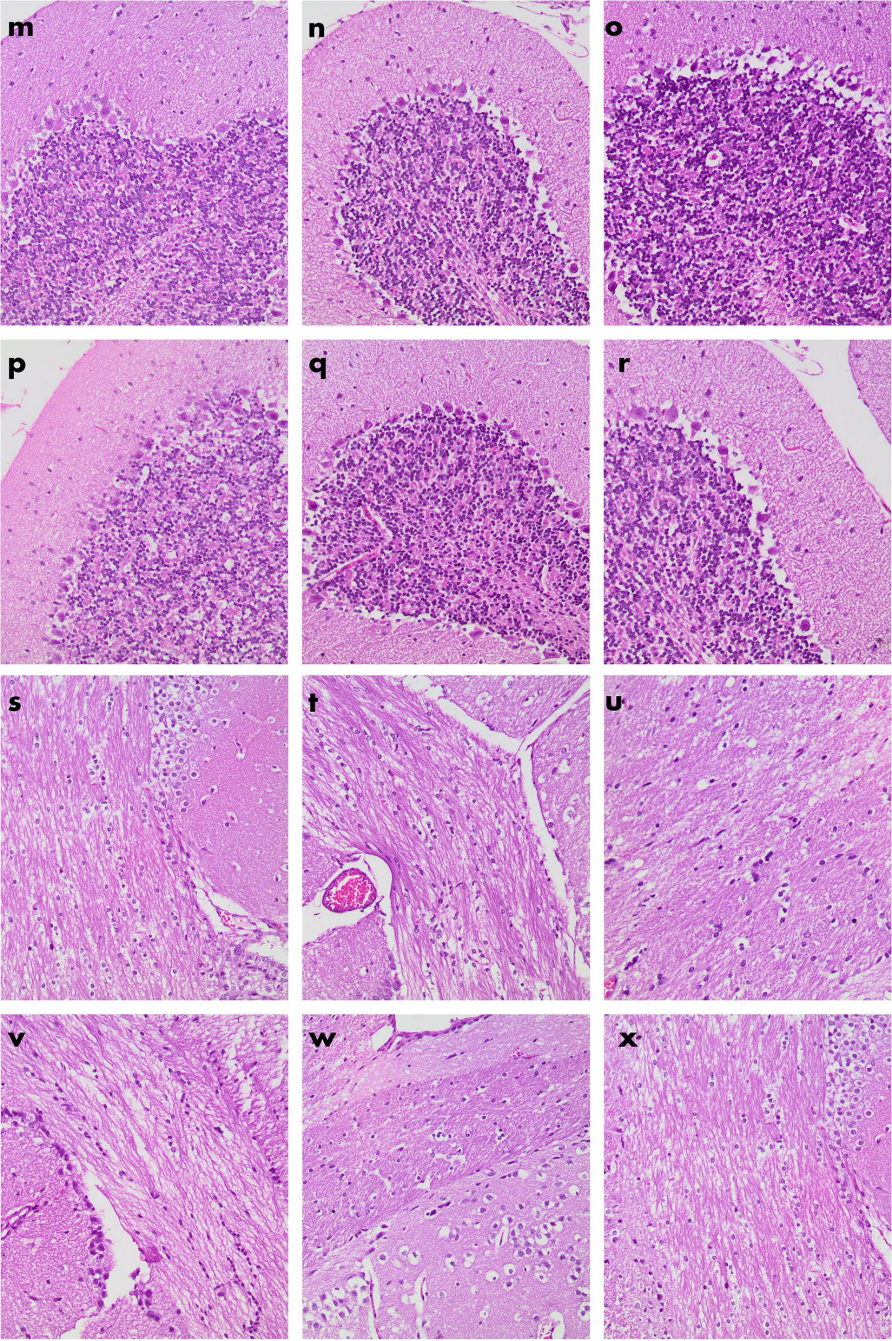
H&E stain of brain tissue of BALB/c mice in all experimental groups. Sections (**a**–**f**) show parts of the cortex of the following groups sham, saffron sham, crocin sham, TBI, saffron TBI, and crocin TBI respectively (4×). Sections (**g**–**l**) show parts of the denta gyrus of the following groups, sham, saffron sham, crocin sham, TBI, saffron TBI, and crocin TBI respectively (10×). Sections (**m**–**r**) show parts of the cerebellum of the following groups, sham, saffron sham, crocin sham, TBI, saffron TBI, and crocin TBI respectively (20×). Sections (**s**–**x**) show parts of the corpus collosum of the following groups, sham, saffron sham, crocin sham, TBI, saffron TBI, and crocin TBI respectively (20×). All sections appear without any neuronal loss and without any axonal spheroids among all groups. No evidence was shown for tissue loss, contusions, edema, or intraparenchymal hemorrhages in sections of all groups. All sections shown were obtained after 7 days from the final injury.

Histological analysis of the brain tissue of all experimental groups is shown in Fig. 5. General morphological examinations in the brain including neuronal injury, cell death, intracranial hemorrhages, and edema formation were assessed. No neuronal loss in the cortex (Fig. 5a–e), dentate gyrus (Fig. 5f–k), and cerebellum (Fig. 5l–q), observed in the TBI group. There were no axonal spheroids in all sections of the corpus callosum (Fig. 5r–x) and no intraparenchymal hemorrhages as well in any sections of all groups.

### Saffron extract and crocin suppress TBI-induced inflammatory and oxidative responses

To determine the protective role of saffron and crocin against inflammation and oxidative stress, the levels of IFN-γ, TNF-α, MPO, GSH, and MDA were measured in the cortex homogenate 24 h post-final injury.

**Figure 6:**
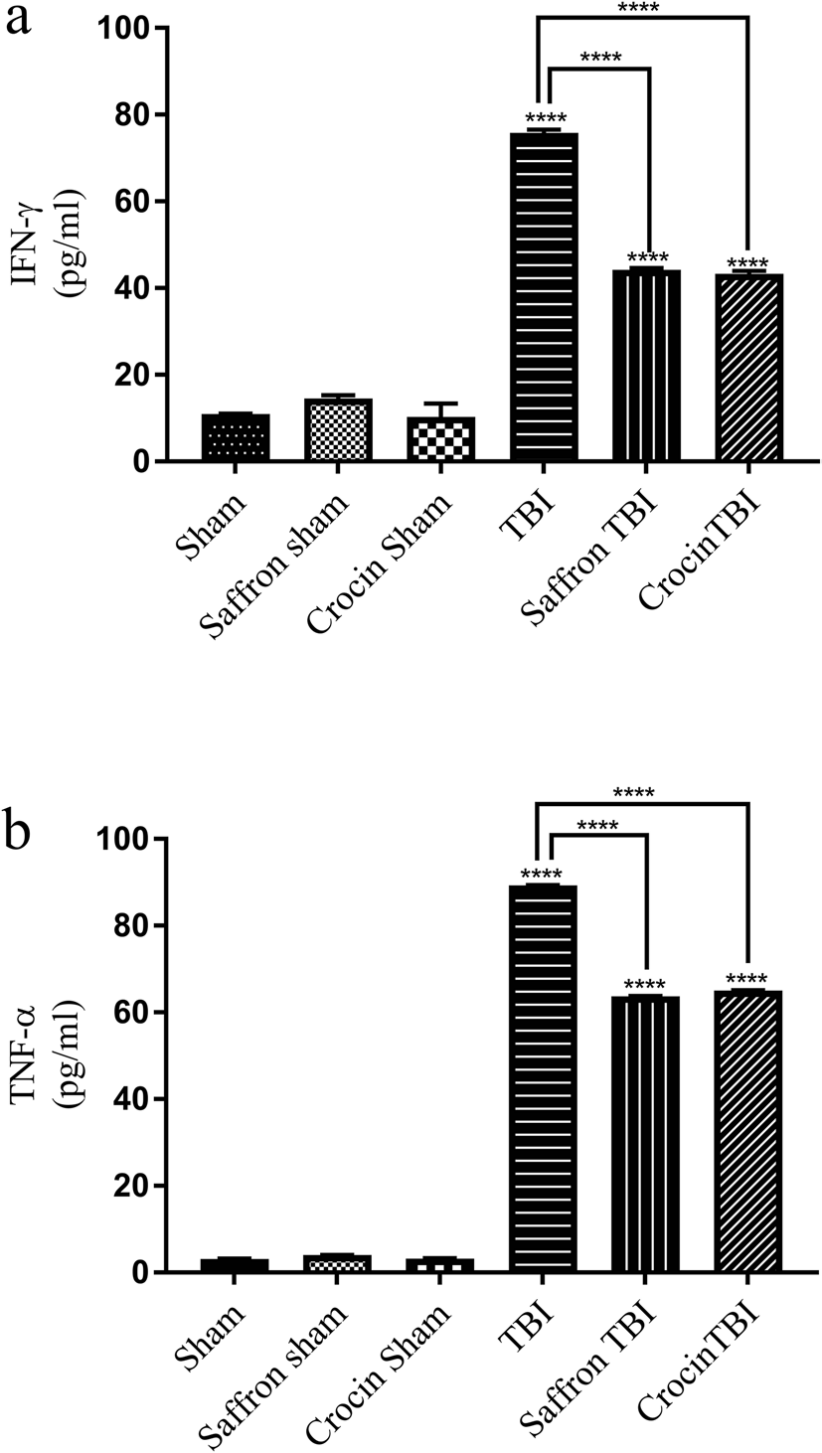
Effect of saffron extract and crocin on IFN-γ and TNF-α Levels in cortex homogenates. (**a**) IFN-γ levels in all groups. (**b**) The concentrations of TNF-α. Data are represented as means of three determinations ± SEM. (****) corresponds to p-value < 0.0001.

IFN-γ levels significantly increased in the TBI group by 7 folds (p < 0.0001) as shown in Fig. 6a. Treatment with saffron extract and crocin induced a notable equal decrease in IFN-γ levels by 1.7 folds (p < 0.0001) as compared to the TBI group. Similarly, the pro-inflammatory cytokine TNF-α increased significantly by 28 folds (p < 0.0001) in the TBI group (Fig. 6b). Treatment with both saffron extract and crocin were able to significantly decrease TNF-α by 1.4 folds (p < 0.0001).

**Figure 7:**
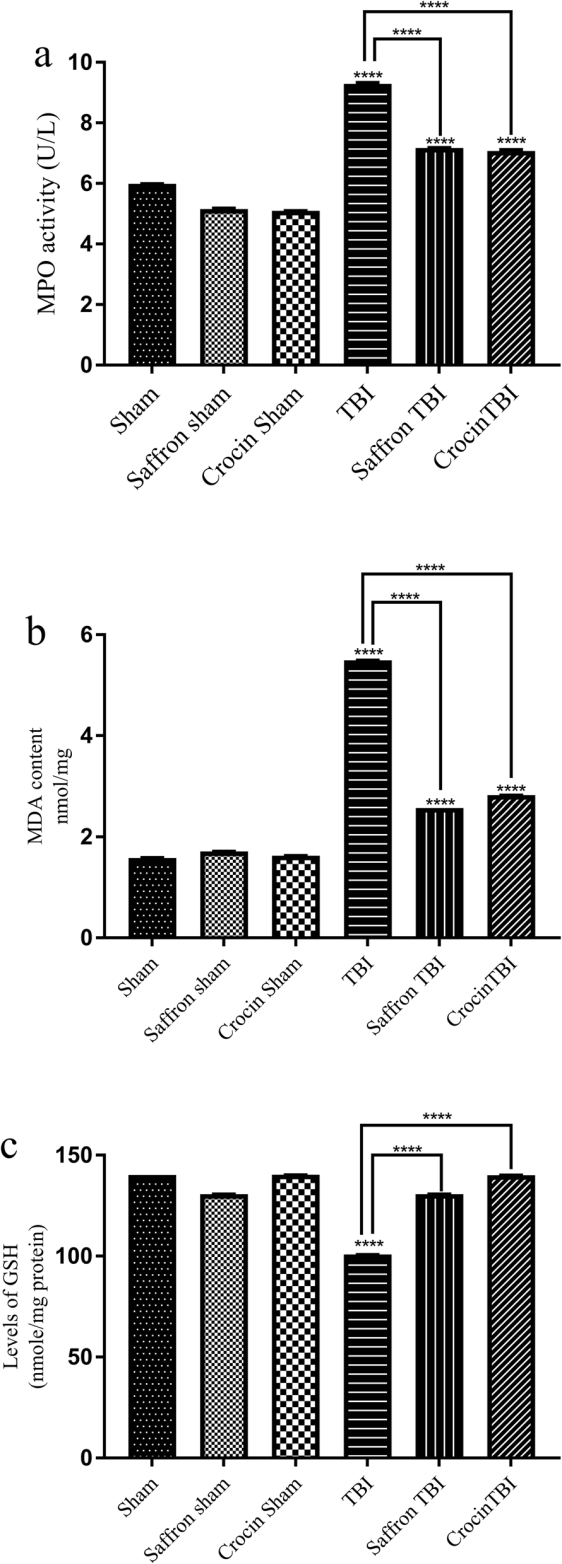
Effect of saffron extract and crocin on MPO activity, MDA content, and GSH levels. (**a**) The significant increase in activity of MPO in TBI, saffron TBI and crocin TBI groups and shows the relative decline in MPO activity in saffron TBI and crocin TBI groups compared to the TBI group. MDA content is expressed in (**b**). The level of MDA is significantly high in the TBI, saffron TBI, and crocin TBI groups. (**c**) GSH levels in the experimental groups. TBI caused a significant decrease in GSH levels while saffron and crocin treatment restored normal GSH levels. Both treatments were significantly effective in reducing MDA levels. Data are represented as means of three determinations ± SD. (****) corresponds to p-value < 0.0001.

The MPO, MDA, and GSH levels in the cortex samples are shown in Fig. 7. The three oxidative indicators demonstrated that rmTBI generated an oxidative stress that was considerably mitigated by both treatments. Brain injury induced a significant increase in MPO levels in TBI group (1.5 folds, p < 0.0001). The increase in MPO levels in the TBI group was attenuated by 1.3 folds after saffron extract and crocin administration (p < 0.0001) (Fig. 7a). As for the MDA levels, the TBI group showed significantly greater levels compared to the sham group by 3.5 folds (p < 0.0001) as shown in Fig. 7b. Saffron extract and crocin were able to reduce the oxidative level as manifested by 2 folds decrease in MDA levels (p < 0.0001). Similar data were obtained when assessing the GSH content which recorded the lowest value in the TBI group with 0.7 folds decrease as compared to the sham group (p < 0.0001) (Fig. 7c). Not only did the administration of saffron extract and crocin increase the GSH levels, but it also restored the normal values comparable to the shams. Given the preceding results, it is believed that the inflammatory and oxidative markers induced by the presented rmTBI model in the cortex tissue were importantly alleviated by saffron extract and crocin treatment. The crocin and saffron administered doses were totally safe and potentially contributed to the amelioration of injury-induced associated behavioral outcomes.

## Discussion

The use of animal models has become indispensable for investigating the pathological and clinical outcomes of TBI, designing therapeutic interventions, and translating them from bench to bed. Today there is more concern for studying mild forms of TBI due to the rising number of patients suffering from mTBI^9^. Although many models were designed to understand rmTBI, there is no universally accepted method to replicate 100% human concussion. As no treatment for TBI has emerged by now, more attention is being driven toward the use of medicinal herbs and plants, many of which possess strong antioxidant and anti-inflammatory characteristics.

In our study, we examined the protective role of saffron and its major bioactive ingredient crocin in a repetitive mild traumatic brain injury mouse model. The non-invasive process of trauma induction involved dropping a weight directly on the intact skull of an anesthetized mouse, whose head was left free to move during the hit. After several trials of changing the height and time interval between hits, the injury protocol consisted of seven multiple hits with an inter-injury interval of 48 h. We remarkably documented that changing the inter-injury interval is correlated with the magnitude of outcomes as revealed by many previous studies^23–25^.

The NSS recorded after 1 and 24 h following the last hit confirmed the mild aspect of our repetitive injury model with no skull fracture and < 5% mortality rate. Such results are consistent with mortality data conferred by a previous study^26^. We must note that this is the first study that investigates the role of saffron and crocin in a rmTBI weight drop model on closed head mice.

No gross pathological injury or neuronal loss was detected in the injured brains and typical H&E stain revealed normal cell morphology and count in all groups after 7 days. Although most of the research work has focused on the long-term effects of brain injury on histological changes, few studies explored the short-term histological effects^27,28^. In one study by Osier et al.; no neuronal death in the hippocampus was observed 7 days after 2 repeated mild closed head injuries^28^. In another study implying 3, 5, and 10 multiple hits, memory deficit observed 24 h post the final injury was not accompanied by any cell loss as revealed by H&E stain^9^.

Regarding motor coordination skills, TBI mice expectedly showed defective skills that sports athletes frequently encounter after sustaining their concussions^29^. Mice performances on rotarod rod and pole climb were improved but not restored to normal in both treated groups. In previous publication^30^ crocin exhibited motor coordination enhancing skills in neurotoxic, hypoxic, and ethanol withdrawal-induced conditions that could be attributed to its potent anti-inflammatory and antioxidant effects^31^. This is not surprising since saffron and crocin have been presented as effective in managing neurodegenerative and nervous system-related diseases clinically^18^ and in animal models^32,33^. Adhesive tape removal test revealed that rmTBI impaired the sensorimotor function in mice. It is more likely that the cumulative effects of the multiple mild hits on the cortex aggravated the sensorimotor outcomes which were significantly reduced by saffron and crocin treatment.

Impaired cognitive skills manifested by difficulties in memory function, attention and multitasking are marked features of repeated concussions^34^. After 24 h from the last hit, we assessed the memory skills of mice using MWM. Our data showed that over 5 consecutive days the latencies to reach the hidden platform decreased in all sham groups, revealing their cognitive ability to learn and memorize using the spatial cues. On the contrary, TBI mice failed to train and memorize as there was no significant decrease in their latencies which suggests a short-term cognitive deficit manifested after the multiple hits. Interestingly, the latencies result of both treated groups were like their corresponding shams, reinforcing previous findings on the role of saffron and crocin in enhancing cognitive deficits^35^. Given that in long-term memory, deficits have been reported in rmTBI clinical studies^21^ and using rodent models^36^. Additional research is required to explain whether saffron and crocin exert the same effects on long term cognitive behavior.

TBI consists of two interdependent phases, the primary injury, and the secondary injury. The primary injury is the mechanical displacement of tissue and usually, at this phase, no treatments are effective^37^. Secondary mechanisms underlain by lipid peroxidation, inflammatory and oxidative stress exacerbate the outcomes after a TBI^38^ along with astrocyte and microglial activation and neuronal injury, the therapeutic window time is crucial for using pharmacologically active compounds that might abrogate such delayed complications^39^. Glial fibrillary acidic protein (GFAP), ionized calcium-binding adaptor molecule 1 (Iba1) are major markers of astrocyte and microglial activity respectively^40,41^ and neuronal nuclei (Neu N) acts as a neuronal damage marker^42^. In our recent study^43^, the expression levels of these markers were measured to assess the head injury and an increase in their expression levels was observed in the TBI group confirming induction of astrocyte, microglial activation, and neuronal injury.

Lipid peroxidation contributes to irreversible deleterious consequences to membrane proteins and lipids, mediated by the accumulation of reactive oxygen and nitrogen species^44^. It has the ability to initiates various neurodegenerative and TBI-related pathologies^45^ such as CTE^46^, MDA, the end product and marker of lipid peroxidation^47^ and GSH, a major intracellular antioxidant source were measured 24 h post last injury^48^. MDA levels were significantly high in the TBI group and this finding aligns with increased MDA levels in brain tissues from TBI patients^49^ as well as in rodent models^48,49^. In saffron TBI and crocin TBI groups, MDA decreased significantly, suggesting that saffron and crocin could relieve the peroxidative damage induced by rmTBI. The oxidative stress beside to GSH. GSH levels were reduced in the TBI group and this outcome is implied in prior TBI study^50^. The marked decline in lipid peroxidation and increase in GSH content with both treatments is attributed to the free radical scavenging potential of saffron and its bioactive carotenoid crocin^51^. Relevantly, as proposed by previous studies^52^, one of the antioxidant mechanisms of saffron and crocin to protect the brain against oxidative stress, neurobehavioral impairment, and aluminum toxicity is decreasing MDA and increasing GSH content.

Different rodent models of single or repetitive injuries have been designed to figure out the divergent pathophysiology of human TBI^53^ and neuroinflammation has always been a consistent complexity detected in all TBI conditions regardless of its severity^54^. The genes involved in neuroinflammation are upregulated post-TBI and can remain elevated for 17 years post-TBI^14^. This reflects the potent role of inflammation in driving the complications that evolve after head injury and lead to neurodegeneration and neurological impairments^55^. Neuroinflammation is usually marked by an increase in neuronal cell death, glial cell activation, excessive release of inflammatory cytokines^14,55,56^, and an increase in myeloperoxidase (MPO) levels^57^. MPO expression in the brain is mostly induced in the presence of diseases^58^ and the results of our study supported this, as MPO levels were only elevated in the TBI group. A noticeable reduction in MPO was observed in saffron TBI and crocin TBI groups which is consistent with previous reports that revealed their role in downregulating MPO, thus attenuating oxidative stress and inflammation^59^. Although the role of MPO in TBI is still not well established^60^, an increase in its gene and serum levels is correlated with cognitive dysfunction. This may justify one of the possible mechanisms conferred by saffron and crocin to enhance cognitive skills^61^ and can explain our obtained results. Over years, there had been robust evidence of the anti-inflammatory role of saffron and crocin in many diseases^61,62^. Inflammation can be a double-edged sword after the TBI onset. Overactivation of inflammatory mediators such as cytokines and inflammasomes may lead to detrimental neurological consequences that can be improved using anti-inflammatory interventions^62^. To assess saffron and crocin anti-inflammatory efficacy following repetitive injury, we measured the levels of brain cytokines TNF-α and IFNγ 24 h after the last brain hit, and in our recent study^43^ we assessed the expression levels of NF-κB, NLRP3, and its related gene caspase-1, the apoptosis-associated speck-like protein containing a CARD (ASC), IL-1β, and IL-18. The obtained data reasonably explains the emergence of cognitive and motor deficits in our model, as inflammation is associated with such elicited acute disturbances following TBI^63–66^. Sirtuin 1 (SIRT1) is also an essential element for normal cognitive function and synaptic plasticity^67^ and proved to enhance cognitive dysfunction in many studies^68,69^. The measured SIRT1 levels were also suppressed post TBI^43^. In alignment with our data, inhibition of NLRP3 inflammasome has shown to play a crucial part in ameliorating the cognitive deficits induced in diabetic rats following ischemia^70^, encephalomyelitis^71^, AD models^68^, and TBI models^72^. It is suggested that saffron extract and crocin treatment improved the behavioral and cognitive deficits by inhibiting the inflammasome pathway, lowering levels of IL-1β and TNF both associated with impaired sensiromotor outcomes^69^, and elevating SIRT 1 expression levels. Our results showed that the levels of TNF-α and IFNγ were higher in the TBI group than the sham levels. Saffron and crocin alleviated the inflammation mediated by these cytokines by reducing their levels in the brain as manifested when comparing saffron TBI and crocin TBI with the TBI group. Decreased TNF-α levels accompanied with neuroprotection against apoptosis, edema, and neurological severity were also reported in the only study that addressed the role of crocin in a CCI TBI model in mice^62^.

Based on our data, it is believed that saffron and crocin can enhance cognitive and motor rmTBI induced acute deficits by reducing lipid peroxidation, increasing GSH, and suppressing inflammatory mechanisms thus protecting them against the subsequent release of reactive oxygen and nitrogen species that are associated with harmful behavioral damages.

In conclusion, our findings present a robust role of saffron and its major ingredient, crocin, in conferring protection against the oxidative and inflammatory complications following concussions. Such data can hopefully contribute to the progress in the field of pharmacological interventions that, along with rehabilitation strategies, aid in targeting long term cognitive and motor sequelae. Promising results would offer insights for more pharmacological therapeutic interventions that target concussion survivors.

## Methods

### Preparation of saffron water extract and crocin

Saffron grade 1 stigmas were purchased was purchased from Novin Saffron Company provided by Mehran Trading Company (Mashhad Iran, ISIC code: 4721). A 2% aqueous solution was prepared by soaking one gram of ground saffron stigmas in 50 ml of distilled water for 3 h in dark. The filtrate was then freeze-dried yielding 0.065 g of a yellow-orange precipitate. The extracted sample was stored in a sealed dark container at − 20 °C. Aliquots of extract residues were weighed and suspended in saline to a final concentration of 8.6% before use. Crocin (42553-65-1) was purchased from Sigma-Aldrich Company (USA) and a 1% solution was prepared in normal saline.

### Experimental groups

Male albino BABL/c mice weighing 30–40 g were obtained from the animal house. They were housed under standard laboratory conditions of light (12 h light/dark cycle), humidity, and temperature and had ad libitum access to standard mouse diet and tap water. Animal experiments were conducted and approved according to the guidelines of the Institutional Review Board (IRB) of Beirut Arab University code number (2019A-0039-S-P-0334). The described study was carried out in compliance with the ARRIVE guidelines. 150 Mice (n = 25 per group, n = 4 per cage) were randomly divided into six groups: sham, saffron sham, crocin sham, TBI, saffron TBI, and crocin TBI. To obtain significant and credible data, eight mice per group were used for behavioral studies and 16 mice per group for molecular and histological analysis. TBI groups were subjected to 7 hits over 14 days with 48 h intervals. Mice were treated either with saffron extract (50 mg/kg) or crocin (30 mg/kg) administered intraperitoneally, 30 min before every hit. The brains’ cortices (n = 3 per group) were removed for histological and molecular studies (n = 13 per group). Each group with n = 25 received the treatment as listed in supplementary Table S1. Any mouse exhibiting fatigue and inflammation signs or any deformation that lead to biased results was excluded from the study. To minimize potential confounders’ effect, each task was performed by the same researcher each time and all cages were returned to their same location. One investigator administered the treatment and the other performed anesthesia and hit induction and monitored the behavior post injury.

### Brain injury induction

**Figure 8:**
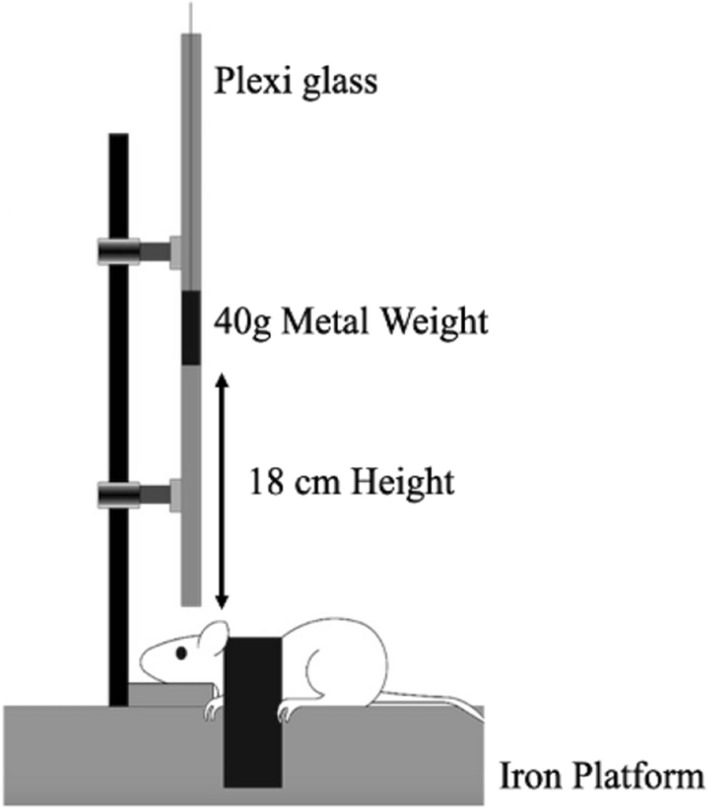
Scheme of TBI apparatus used to induce brain trauma.

The rmTBI injury setup was developed using a weight drop model mimicking closed head injury. The model is schematized in Fig. 8. A 40 g metal of 10 mm diameter was freely dropped from 18 cm height through a 50 cm plexiglass tube. Animals were anesthetized by an intraperitoneal injection of a combination of xylazine (10 mg/kg, Interchemie Weken, Holland) and ketamine (50 mg/kg, Sterop Belgium). Untreated and treated TBI mice were placed on an iron platform without head fixation to cause a diffuse injury. The injury was repeated 7 times for 14 days with a 48 h interval. Mice were injected either with saffron extract (50 mg/kg) or crocin (30 mg/kg) intraperitoneally 30 min before every hit. After receiving the brain injury, mice were laid on heat pads for 10 min before being returned back to their home cage.

### Behavioral assessment

Eight mice from each group were used for blinded behavioral studies 24 h after the final injury. Mice were euthanized by cervical dislocation after completion of behavioral studies. The experimental timeline is shown in Supplementary Fig. S1 online.

#### Neurological severity score (NSS)

For neurologic al assessment, a 10-point neurological severity score (NSS) was followed as described by Zhuang et al.^73^. The test included motor (muscle status and abnormal movement), sensory (visual, tactile, and proprioceptive), reflex, and balance tasks assigned for each mouse. The NSS was assessed at 1 h and 24 h post the final injury. The difference between the NSS at two different time points is a parameter that reflects injury severity post-TBI, as described by Chen et al.^74^. The scores were classified as follows: 13–18 severe injury, 7–12 moderate injury, 1–6 mild injury. A concise overview of the NSS followed can be found as Supplementary Table S2 online.

#### Morris water maze (MWM)

MWM test was performed 24 h after the final injury based on a previously described method by Hamm et al.^75^. The paint was added to turn the color turbid. A translucent platform (diameter 15 cm, and height 30 cm) was submerged 1 cm below the surface of the water. Visual cues of black and white geometric designs were hung on the wall of the tank. MWM experiment was done to evaluate the short-term effects of TBI on memory. Latency to find the platform was measured over 5 consecutive days. Mice were subjected to four trials on day 1, three trials on days 2, 3, 4, and one trial on day 5. Mice that failed to find the platform within 60 s were removed and placed on the platform for 15 s before being returned to their cages. Videos of the whole process were taped and results interpreted using Graph pad Prism.

#### Rotarod and pole climb test

Rotarod and pole tests were used to evaluate motor dysfunction as described by Bouet et al.^76^. In both tests, mice were trained before evaluation. The rotarod was set to start at an initial speed of 4 rpm and accelerate to 40 rpm gradually over 300 s. Each mouse performed the task three times at each setting and the latency to fall was recorded. Each trial on the rod was terminated when the animal fell off the rod or remained on the rod for 300 s. For the pole test, mice were placed on the top of a 70 cm vertical pole with a diameter of 1 cm. The pole was mounted on a rectangular base stand and placed in the home cage so that mice might prefer to descend to the floor of the cage. Time to cross half the rod and time to reach the cage floor were recorded. If the animal paused while descending, the trial was repeated. To avoid sliding, the surface of the pole was rolled with tape. The test was repeated three times for each animal.

#### Adhesive test

A modified adhesive test^77^, was performed following the rotarod test. A tape was placed on the mouse's nose and then the mouse was returned to its home cage. The latencies to touch and remove the tape with one of the forepaws were measured. Each trial was terminated after 1 min if the tape was not removed. The test was repeated three times and the cage was cleaned with ethanol after each trial.

### Histological and biochemical analysis

For histopathological analysis, animals were anesthetized and sacrificed 7 days post the final injury and perfused through the aorta with 10% formaldehyde in phosphate buffer (pH 7.4). After decapitation, the brains of mice were fixed in the same fixative for 24 h at room temperature. Tissue blocks were sectioned 6 μm thickness using a microtome (14274 microtome HM340E), deparaffinized, and stained with Hematoxylins and Eosin. The sections were examined with a Zeiss Primo light microscope with Axio Vision software (400× magnification) and photomicrographed using AxioCam camera for analysis.

For biochemical analysis, mice were anesthetized with ketamine and xylazine and sacrificed 24 h post the final injury. The cortices were collected and immediately frozen in liquid nitrogen and stored at -80 °C until use. Brain cortices were mechanically homogenized in lysis buffer (10 mM PBS containing 1 mM PMSF) at ratio 1:9 (w/v) and then centrifuged for 5 min at 5000 × *g*. The supernatants were subsequently used for MDA analysis^78^ and cytokines analysis. Total protein concentration was determined using the Bradford protein assay^79^.

### Cytokines levels

The levels of pro-inflammatory cytokines TNF alpha (TNF-α) and interferon-gamma (IFN-γ) were measured using commercially available kits (Elabscience, United States, catalog number: E-EL-M0049 for the TNF-α and E-EL-M0048 for IFN-γ) according to the manufacturer’s instructions. The experiments were conducted as described in our previous work^43^. In brief, standards and supernatants of homogenates were added to the 96-well plates coated with the specific murine monoclonal antibodies raised against TNF-α or IFN-γ. Plates were then incubated for 90 min at 37 °C followed with gentle decantation and without any washing, the biotin-labeled specific antibodies for TNF-α and IFN-γ were added and incubated for 60 min at 37 °C. Following this incubation, wash with the kit washing buffer before applying the HRP conjugate for 30 min at 37 °C and after that. Finally, the substrate reagent was added and the plates were incubated for 15 min at 37 °C beforehand stopping the reaction and reading the absorbance of the developed color at 450 nm. The inflammatory cytokine contents in the brain tissues were expressed as pg/ml.

### Determination of the antioxidant indices

Tissue levels of the antioxidant indices glutathione (GSH) and myeloperoxidase activity (MPO) were measured 24 h post the final injury by commercial kits (Elabscience, United States, catalog number E-BC-K051 for GSH and E-BC-K074 for MPO) according to the instruction of the manufacturer. The levels of malondialdehyde (MDA) were determined according to Buege and Aust^80^.

### GSH assay

In brief, the cortical tissue was weighed and mechanically homogenized in lysis buffer (PBS 0.01 M, pH7.4) at a ratio of 1:19 (w/v), in an ice bath, and then centrifuged at 10,000 × *g* for 15 min. The supernatant was collected for spectrophotometric measurement of GSH according to the manufacturer's instructions. Di-thio-dinitro-benzoic acid is used to calculate the GSH content indirectly expressed as mg per g of protein.

### Myeloperoxidase assay

For measuring myeloperoxidase (MPO) activity, the cortex mass was accurately weighed and mechanically homogenized in an ice bath with buffer solution provided with the kit at a ratio of 1:19 (w/v). The myeloperoxidase reduces hydrogen peroxide to a complex that will react with o-dianisidine (as hydrogen donor) to produce a yellow product which has a maximum absorption peak at 460 nm. The activity of MPO was expressed as U/l.

### Malondialdehyde assay

Malondialdehyde (MDA) levels were determined using the thiobarbituric acid reactive substance assay (TBARS). The brains were homogenized with lysis buffer at a 1:9 ratio (w/v). Homogenates (50 µl) were mixed with thiobarbiturate TBA solution (100 µl, 0.5% TBA in 20% TCA) and boiled for 20 min for chromophore development according to Buege and Aust^80^. Samples were placed in a 96 well plate and absorbance was read at 532 nm. The MDA content was calculated according to the following equation:

$$\begin{aligned} {\text{MDA }}\left( {\text{M}} \right) = ({\text{A sample X DF}})/{\text{l}} \times \varepsilon \end{aligned}$$

where the extinction coefficient $\varepsilon {\text{ is 1}}.{56} \times {1}0^{{5}} \;{\text{M}}^{{ - {1}}} \;{\text{cm}}^{{ - {1}}} .$ MDA is expressed in nmol/mg protein

### Statistical analysis

The analysis was performed using the Graph Pad Prizm. The biochemical analysis and all behavioral tests except the MWM were analyzed by one-way repeated measure ANOVA followed by the Bonferroni test. For MWM data analysis, 2-way ANOVA was used followed by Tukey’s test. Weight analysis was analyzed using one-way repeated measure ANOVA followed by Tukey’s test. All results were expressed as the mean ± SEM. A level of p < 0.05 was considered statistically significant.

### Ethics declarations

Animal experiments were conducted and approved according to the guidelines of the Institutional Review Board (IRB) of Beirut Arab University code number (2019A-0039-S-P-0334). The described study was carried out in compliance with the ARRIVE guidelines.

## Supplementary Information

Supplementary Information 1.

Supplementary Information 2.

Supplementary Information 3.

## Data Availability

The datasets used and/or analysed during the current study are available from the corresponding author on reasonable request.
