# Supplementary figures and images for: Saffron extract and crocin exert anti-inflammatory and anti-oxidative effects in a repetitive mild traumatic brain injury mouse model
Source: Sci Rep. 2022 Mar 23;12:5004. doi: 10.1038/s41598-022-09109-9 (PMC8943204; doi:10.1038/s41598-022-09109-9)

**Supplementary Fig. S1. Experimental timeline**

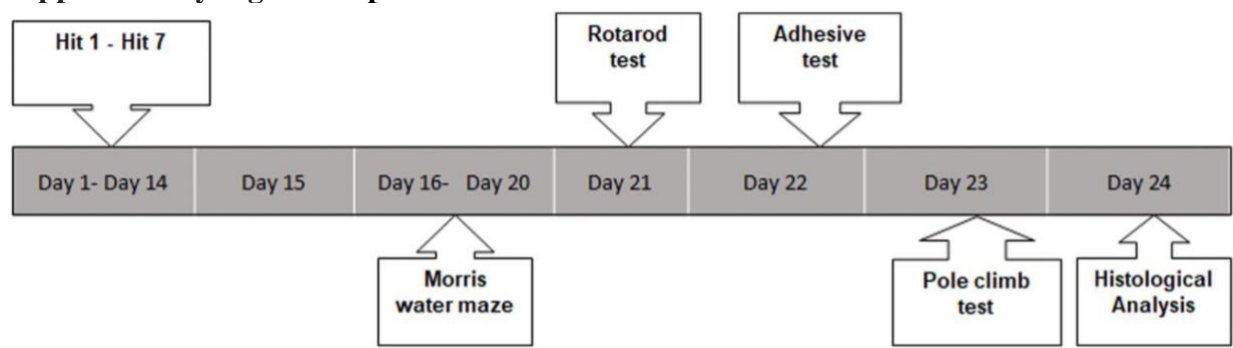

Supplement: Supplementary file 1 — Supplementary Information 1. [file 41598_2022_9109_MOESM1_ESM.pdf]
