# Supplementary material for: Saffron extract and crocin exert anti-inflammatory and anti-oxidative effects in a repetitive mild traumatic brain injury mouse model
Source: Sci Rep. 2022 Mar 23;12:5004. doi: 10.1038/s41598-022-09109-9 (PMC8943204; doi:10.1038/s41598-022-09109-9)
Supplement: Supplementary file 2 — Supplementary Information 2. [file 41598_2022_9109_MOESM2_ESM.pdf]

Supplementary Table S1. Experimental groups and treatment protocol.

Groups	Treatment	Dose
Sham	Anesthesia (Intraperitoneal injection)	Xylazine (15 mg/kg) combined with ketamine (50mg/kg)
TBI	Anesthesia (Intraperitoneal injection) + 7 injuries	Xylazine (15 mg/kg) combined with ketamine (50mg/kg)
Saffron sham	Saffron (Intraperitoneal injection)	Xylazine (15 mg/kg) combined with ketamine (50mg/kg) +50mg/kg Saffron
Saffron TBI	Saffron (Intraperitoneal injection) +7 injuries	Xylazine (15 mg/kg) combined with ketamine (50mg/kg) +Saffron (50 mg/kg)
Crocin sham	Crocin (Intraperitoneal injection)	Xylazine (15 mg/kg) combined with ketamine (50mg/kg) +30mg/kg Crocin
Crocin TBI	Crocin (Intraperitoneal injection) + 7 injuries	Xylazine (15 mg/kg) combined with ketamine (50mg/kg) +30mg/kg Crocin
