# Supplementary material for: Saffron extract and crocin exert anti-inflammatory and anti-oxidative effects in a repetitive mild traumatic brain injury mouse model
Source: Sci Rep. 2022 Mar 23;12:5004. doi: 10.1038/s41598-022-09109-9 (PMC8943204; doi:10.1038/s41598-022-09109-9)
Supplement: Supplementary file 3 — Supplementary Information 3. [file 41598_2022_9109_MOESM3_ESM.pdf]

Supplementary Table S2. Tasks assigned for the neurological severity score.

Task	NSS
Failure to exit a 30-cm-diameter circle 1 for 2 min	1
Presence of monoparesis or hemiparesis	1
Failure of flexion of hindlimb after raising rat by the tail	1
Failure of flexion of forelimb after raising rat by the tail	1
Failure of head moved >10° to vertical axis within 30 seconds after raising rodent by the tail	1
Inability to walk on a 3-cm-wide beam	1
Inability to walk on a 2-cm-wide beam	1
Inability to walk on a 1-cm-wide beam	1
Failure to Balances with steady posture om 0.5cm beam balance	1
Hugs beam and 1 limb falls from the beam (0.5cm wide)	1
Hugs beam and 2 limbs fall from the beam or spins on beam (60 seconds)	1
Attempts to balance on the beam, but falls off (> 40 seconds)	1
Attempts to balance on the beam, but falls off (> 20 seconds)	1
Falls off; no attempt to balance or hang on to beam (< 20 seconds)	1
Absence of pinna reflex	1
Absence of corneal reflex	1
Absence of startle reflex	1
Failure to have a straight walk	1
